# Workplace suspicion, knowledge hiding, and silence behavior: A double-moderated mediation model of knowledge-based psychological ownership and face consciousness

**DOI:** 10.3389/fpsyg.2023.982440

**Published:** 2023-03-15

**Authors:** Mengying Wu, Wei Li, Lei Zhang, Chi Zhang, Hanhui Zhou

**Affiliations:** ^1^College of Philosophy, Law and Political Science, Shanghai Normal University, Shanghai, China; ^2^Glorious Sun School of Business and Management, Donghua University, Shanghai, China; ^3^Kedge Business School, Marseille, France; ^4^Business School, Huaqiao University, Quanzhou, China

**Keywords:** workplace suspicion, knowledge hiding, silence behavior, knowledge-based psychological ownership, face consciousness

## Abstract

Silence behavior is a common and influential phenomenon in organizations. Scholars have explored a lot of antecedents for silence behavior, but rarely from the perspective of colleagues. Based on the conservation of resources theory and self-regulation theory, the study constructs a double-moderated mediating model to explore the relationship between workplace suspicion and silence behavior as well as its mechanism. This study conducts a three-wave questionnaire survey and adopts 303 valid pairs of samples from 23 companies in China to validate the research hypotheses. A confirmatory factor analysis in the AMOS software and the PROCESS bootstrapping program in SPSS is used in this study. Our findings indicate that workplace suspicion is positively correlated with silence behavior; knowledge hiding mediates the relationship between workplace suspicion and silence behavior; knowledge-based psychological ownership moderates this mediating effect by strengthening the negative impact of workplace suspicion on knowledge hiding; and face consciousness moderates the mediating effect by weakening the positive impact of workplace suspicion on knowledge hiding. Managerial and practical implications, limitations, and future research directions are discussed and offered.

## 1. Introduction

In today’s era of increasingly complex business and fierce competition environment (Roscoe et al., 2020; Lang et al., 2022), as the basic elements of the company, employees play an exceedingly critical role in the discovery, opinions, and suggestions of issues (Ng and Feldman, 2012; Liu et al., 2020; Song et al., 2021). However, a large number of employees are reluctant to speak up and choose to remain silent (Milliken et al., 2003; Prouska and Psychogios, 2018). In an interview with employees of an American high-technology corporation, Detert and Edmondson (2011) found that approximately 50% of the interviewees felt uncomfortable speaking up and were extremely sensitive to the problems of the enterprise or work. This phenomenon is frequently observed and is called “employee silence” (Dyne et al., 2003; Brinsfield, 2013), and it can exert destructive impacts on both organizations and individuals. In terms of organizations, employee silence could reduce the quality and efficiency of organizational decision-making as well as be a critical barrier to organizational change and development (Morrison and Milliken, 2000; Dyne et al., 2003). Regarding individuals, as a significant demoralizing force (Srivastava et al., 2019), employee silence could generate stress (Morrison and Milliken, 2000; Xue and Yang, 2021), and job burnout (Srivastava et al., 2019), thereby decreasing innovative work behavior (Guo et al., 2018; Maqbool et al., 2019) and task performance but increasing deviant behavior (Xue and Yang, 2021). Therefore, how to manage employee silence behavior has attracted the extensive attention of scholars and practitioners.

Currently, a large number of studies have confirmed the influencing factors of employee silence from different perspectives, providing great help for organizations to understand and manage employee silence, including individual factors such as individual cognition (Yan et al., 2022), personality differences (Timming and Johnstone, 2015; Wan et al., 2021), and self-esteem level (Duan et al., 2018); leadership factors such as leadership style (Li and Xing, 2021; Wei et al., 2022) and leader-member exchange relationship (Xu et al., 2015; He et al., 2022); organizational factors such as organizational structure (Wynen et al., 2020), atmosphere (Tangirala and Ramanujam, 2008; Wang and Hsieh, 2013; Zhu and Xie, 2018), and politics (Jaweria and Jaleel, 2016); and other factors. However, whether employees will remain silent depends not only on their own leadership and organizational factors but also on the influence of colleagues, because the behaviors and attitudes of employees will inevitably be influenced by their colleagues in the same group (Schulte et al., 2012; Chen et al., 2021). Consequently, it is particularly necessary to find the reasons for employees’ silence among colleagues. Recent empirical studies show that employees could respond to negative behaviors (e.g., ostracism and bullying) in the workplace by keeping silent (Rai and Agarwal, 2018; Liu et al., 2020; Yao et al., 2022). Similarly, will workplace suspicion, a relatively obscure negative behavior among colleagues (Bobko et al., 2014; Zhou et al., 2017), affect employee silence behavior? The focus on workplace suspicion could thus expand our understanding of the reasons for silence behavior in the organization.

While the relationship between workplace suspicion and colleagues’ silence behavior has been ignored, research about the underlying mechanism through which workplace suspicion is associated with colleagues’ silence behavior is even scarce. To solve the above problems, this study attempts to explore the influence of colleagues’ suspicion on employees’ silence behavior in the workplace from the perspective of resource conservation theory (COR). The COR theory indicates that individuals strive to maintain valued resources to protect themselves from further resource loss when facing a threatened or actual loss of resources (Hobfoll and Stevan, 1989). Drawing on the COR theory, suspicion perceivers (i.e., employees who suspect their colleagues) could consume a lot of cognitive resources when they suspect the targets (Fein, and Steven., 1996) and then may adopt knowledge hiding as a resource-protecting strategy (Feng and Wang, 2019). In addition, when colleagues engage in knowledge-hiding behavior, employees will remain silent as a psychological breach of contract (Bari et al., 2020). Inspired by this, we found “a key,” knowledge hiding, to open the “black box” of the relationship between workplace colleagues’ suspicion and silence behavior. Knowledge hiding may afford a circumstance for the suspicious targets to explain and attribute the suspicious behavior of the suspicion perceivers.

However, not all employees will show the same degree of behavioral response when facing workplace suspicion. The COR theory points out that individuals’ responses to resource loss associated with workplace stressors are contingent on their characteristics and differences (Hobfoll and Shirom, 2001). Knowledge-based psychological ownership makes an individual psychologically keep some particular knowledge and regard it as the extension of personality (Khalid et al., 2019), which functions as an accelerator in knowledge hiding (Peng, 2013). Apart from that, self-regulation theory holds that individual behavior is affected by behavioral expectations and social expectations to a certain extent (Baumeister et al., 2005). Face consciousness as a human, universal in nature (Ho, 1976) seems more salient in collectivistic societies like China and has a profound impact on individual behavior (Zhang et al., 2011; Zheng et al., 2017; Jin et al., 2022). Therefore, we further expand our research model by examining whether knowledge-based psychological ownership and face consciousness alleviate or reinforce the effect of workplace suspicion on knowledge hiding, even on colleagues’ silence behavior.

**Figure 1:**
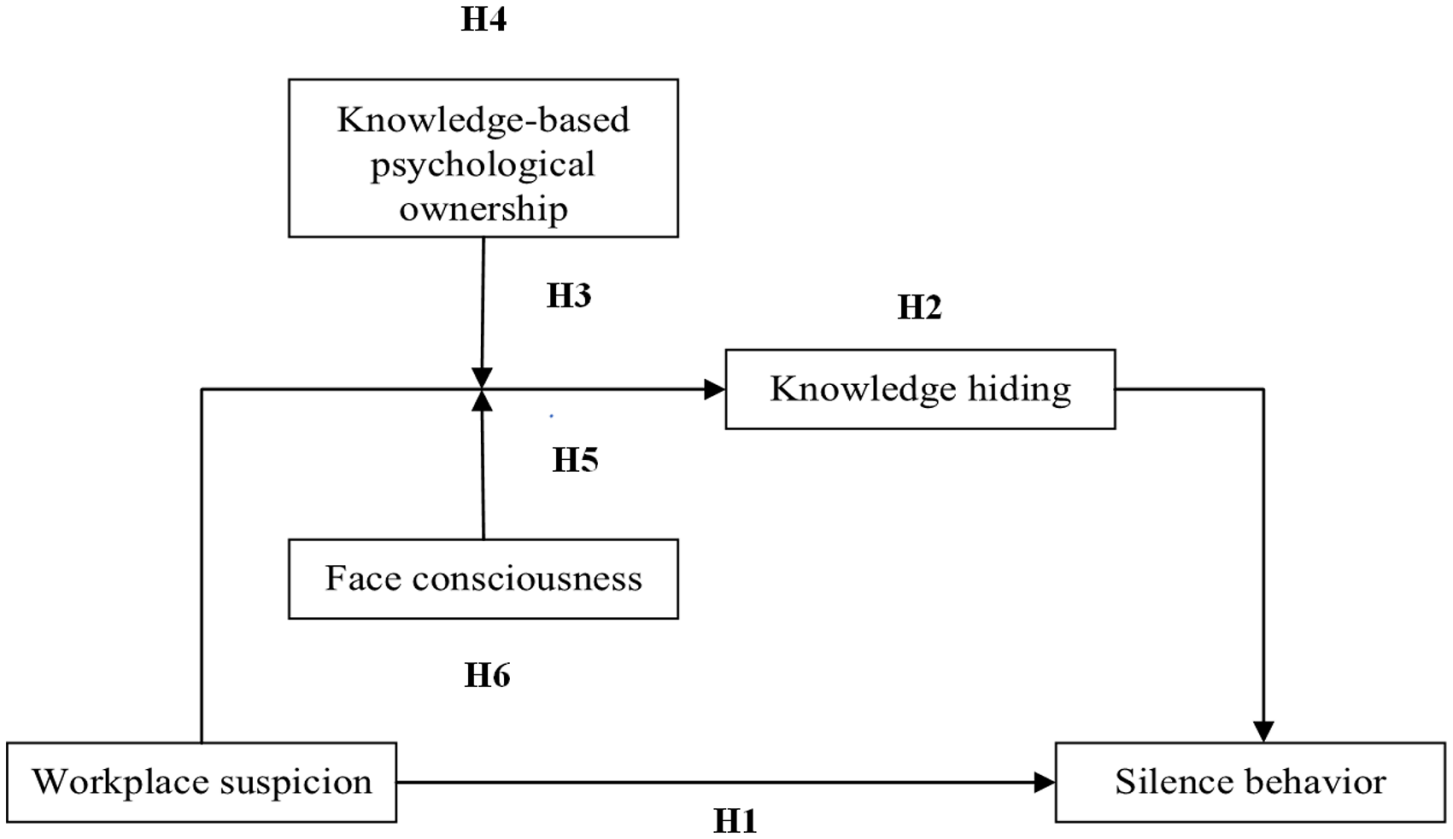
Overall theoretical model.

In summary, this study explores the influence of workplace suspicion on colleagues’ silence behavior, focusing on the mediating effect of knowledge hiding and the moderating effect of knowledge-based psychological ownership and face consciousness (see Figure 1 for the overall theoretical model). The research makes some contributions. First, it extends our present knowledge regarding the antecedents of silence behavior from the perspective of colleagues. Second, by relating suspicion to knowledge hiding and colleagues’ silence behavior, we answer the call of Bobko et al. (2014) for embedding the concept of suspicion in research on business and applied psychology. Third, we test the explanatory mechanism through which workplace suspicion instigates the suspicion targets to stay silent by examining the intermediate role of knowledge hiding. Finally, the study provides new insights into the boundary conditions of workplace suspicion influencing colleagues’ behavior.

## 2. Literature review and hypotheses

### 2.1. Workplace suspicion and silence behavior

Drawing heavily upon Bobko et al.’ (2014, p. 336) definition of state suspicion, we define workplace suspicion (WS) as *an employee’s simultaneous state of cognitive activity, uncertainty, and perceived malintent about other employees and underlying information.* Suspicion, a unique construct, often emerges as a mindset that is neither trusting nor distrusting (Knox, 1970; Fein, and Steven., 1996). Specifically, unlike distrust (Marwan, and Sinaceur., 2010) and other types of interpersonal conflict in the workplace, workplace suspicion has its own unique characteristics. First, workplace suspicion is a perceiver variable, which means it is a kind of personal subjective feeling. Second, as a form of interpersonal conflict, workplace suspicion is implicit rather than explicit. Finally, the definition of workplace suspicion encompasses a number of key elements such as cognitive activity, uncertainty, and perceived malintent, and these elements must be present at the same time.

Employee silence behavior refers to a deliberate intention to withhold ideas, information, opinions, or questions about the job or organizational possible issues and improvements (Dyne et al., 2003). Scholars emphasize that only when employees consciously withhold their views can it be called silence, but the situation of no idea and no voice is not included (Knoll and Dick, 2013). Dyne et al. (2003) propose four dimensions, namely, acquiescent silence, opportunistic silence, defensive silence, and pro-social silence of employee silence behavior, and many other scholars have divided it into acquiescent silence, defensive silence, and indifferent silence (Morrison and Milliken, 2000). An employee may apply one or more of these strategies to remain silent. However, regardless of which path they take, the same result is that they deliberately keep silent in the organization. Based on this view, this study follows the previous research (Tangirala and Ramanujam, 2008), paying attention to the overall level of silence behavior.

According to the COR theory (Hobfoll and Stevan, 1989), this study believes that employees may adopt an avoidance-oriented coping behavior, such as silence behavior at work, to deal with the suspicion of colleagues so as to prevent further loss of resources and retain their remaining resources. The COR theory (Hobfoll and Stevan, 1989) points out that individuals have the motivation to protect their valuable resources and obtain new resources to help them achieve their own goals. Moreover, when faced with pressure sources, individuals’ protection of existing resources is more prominent than the acquisition of new resources (Ng and Feldman, 2012). Academic research suggests that a suspicion perceiver usually takes a more distant and indifferent approach to the suspicion target (Fein and Hilton, 1994). The suspicion perceiver may reduce involvement in the form of nonimmediacy, inexpressiveness, nervous activity, or rigid, overcontrolled behavior that disrupts conversation management (Burgoon et al., 1996). Accordingly, workplace suspicion makes it difficult for colleagues to communicate deeply, which imperceptibly aggravates the consumption of psychological resources by suspicion targets. In the face of this chronic stressor of workplace suspicion, suspicion targets tend to remain silent to protect and observe their limited resources (Hobfoll and Shirom, 2001) and prevent further loss of resources and the adverse effects of suspicion, rather than engaging in more extra-role behaviors to obtain new resources. Therefore, we presume the following hypothesis:

*Hypothesis 1*: Workplace suspicion is positively related to silence behavior.

### 2.2. The mediating role of knowledge hiding

The research suggests that knowledge hiding mediates between workplace suspicion and colleagues’ silence behavior. Connelly et al. (2012) defined knowledge hiding as “an intentional attempt by an individual to withhold or conceal knowledge that has been requested by another person” (p. 65). Unlike silence behavior, knowledge hiding occurs when an individual is requested to share knowledge. Connelly et al. (2012) also expressed that knowledge hiding as a kind of subjective behavior includes rationalized hiding, evasive hiding, and intentional hiding. It is worth mentioning that we also focus on the overall level of knowledge hidden in this article.

Drawing upon COR theory, individuals will strive to protect and obtain resources when they are faced with an actual or threatening loss of resources (Hobfoll and Stevan, 1989). Suspicion triggers an active, attributional thinking that leads the perceiver to elaborate on the true motive for the target’s action, attributing possible negative motives (Fein and Hilton, 1994; Fein, and Steven., 1996; Kim et al., 2004; Bobko et al., 2014). In the process, the relatively large amount of cognitive resources devoted to attributional analyses may tax perceivers’ resources (e.g., energy, time) needed for other tasks (Fein and Hilton, 1994; Lyons et al., 2011). In this situation, suspicion perceivers will become defensive and attempt to conserve remaining resources (Hobfoll and Shirom, 2001). Specifically, when facing requests from their colleagues, they are very unlikely to spend extra time and energy on knowledge sharing to avoid resource loss or further consumption but engage in knowledge hiding instead. In addition, given that suspicion perceivers are in a state of uncertainty and perceived malintent about others (Connelly et al., 2012), they could think mindfully that suspicion targets may be a threat to themselves in the near future. Furthermore, knowledge, as an important individual resource (Hobfoll and Stevan, 1989; Connelly et al., 2012), is also the fear of being threatened. Thus, drawing on COR theory, knowledge hiding may work as a coping strategy in order to ensure that there are sufficient resources to resist potential threats (Wheeler et al., 2010). According to the above, the research posits that workplace suspicion is an important indicator to predict knowledge hiding.

Furthermore, to explain why knowledge hiding leads to colleagues’ silence behavior, we also refer to COR theory (Hobfoll and Stevan, 1989), which suggests that individuals tend to maintain, conserve, and acquire resources. Although interpersonal relationships should be one of the most important sources of employees’ condition resources, knowledge-hiding behaviors directly cause them to deteriorate (Connelly et al., 2012; Connelly and Zweig, 2015). Focal employees suffering from hidden knowledge lose their psychological resources instead of being supplemented by resources from colleagues. In this case, focal employees have insufficient work resources (a lack of support from colleagues), which may aggravate their concerns about the risk of employee voice and make them have serious negative expectations for the results of employee voice, and are more inclined to remain silent. Besides, due to the extra time and energy required for advice, focal employees suffering from knowledge hiding are difficult to willing to make behaviors that may lead to the loss of resources again and are more likely to adopt avoidance-oriented coping behaviors (e.g., silence behavior) to maintain remaining resources. In line with the idea, the existing literature describes that when colleagues feel that they have been denied knowledge, they will also avoid offering suggestions, opinions, and guidance and keep silent (Bari et al., 2020; Jin et al., 2023).

In summary, the above discussions on the influence of workplace suspicion on knowledge hiding and the impact of knowledge hiding on silence behavior suggest that knowledge hiding can afford a circumstance for the suspicion target to explain and attribute the suspicion perceiver’s suspicion. Therefore, we presume the following hypothesis:

*Hypothesis 2*: Knowledge hiding mediates the relationship between workplace suspicion and silence behavior.

### 2.3. The moderating role of knowledge-based psychological ownership

Previous literature has indicated that individuals’ reactions toward workplace stressors may vary in degree (Rai and Agarwal, 2018; Liu et al., 2020). The COR theory emphasizes that individuals’ responses to resource loss associated with workplace stressors are contingent on their individual characteristics and differences (Hobfoll and Shirom, 2001). Psychological ownership that focuses on knowledge represents such an individual characteristic. More specifically, knowledge-based psychological ownership refers to “a state in which individuals feel as though the knowledge of ownership or a piece of knowledge is ‘theirs’ (i.e., ‘It is mine!’)” (Pierce et al., 2001, p. 299; Peng, 2013, p. 400). Employees have personal control over knowledge because it is viewed as a principal source of bargaining power in organizations (Peng, 2013). It is likely that workplace suspicion can result in knowledge hiding, but the extent to which workplace suspicion results in knowledge hiding will be a large function of knowledge-based psychological ownership.

As already outlined, researchers suggest that psychological ownership has frequently emerged as one of the factors that is able to influence individual attitudes and behaviors (Dyne and Pierce, 2004; Peng, 2013; Butt, 2021). Knowledge-based psychological ownership may increase the motivation for knowledge hiding (Peng, 2013). More precisely, employees with high knowledge-based psychological ownership could pay more attention to the knowledge and knowledge value, and they are likely to take control of the knowledge that they view as personal property rather than disclose and transfer it (Gao and Riley, 2010; Peng, 2013; Huo et al., 2016). Brown et al. (2014) have confirmed that individuals with psychological ownership over specific aspects (e.g., knowledge) are inclined to control that knowledge and unwilling to share it with others. Similarly, as suggested by Huo et al. (2016), psychological ownership will increase the territorial nature of knowledge and thus accelerate knowledge hiding.

By extension, the positive relationship between workplace suspicion and knowledge hiding should be strengthened at a higher level of knowledge-based psychological ownership. Previously, we proposed that when workplace suspicion increases from low to high levels, employees become more and more involved in knowledge hiding because suspicion consumes their psychological resources and threatens their real resources (e.g., knowledge). This effect will be more pronounced among those with high levels of knowledge-based psychological ownership. First, as suggested by Ghani et al. (2020), individuals with higher psychological ownership are inclined toward the target, so they are deliberate and thoughtful in their reactions to workplace stressors. Knowledge-based psychological ownership enables individuals to psychologically retain some specific knowledge and regard it as an extension of personality, so as to obtain a sense of esteem, protection, and efficacy from it (Peng, 2013; Qin et al., 2021). By a logical extension of these points, employees with high knowledge-based psychological ownership attach more importance to knowledge value, carry out more cognitive activities, and think about uncertainty, thus increasing knowledge hiding when facing the pressure of their own suspicion. Meanwhile, the sense of possession and control of knowledge makes employees always vigilant against external threats. Therefore, when workplace suspicion moves from low to high levels, employees with higher knowledge-based psychological ownership strongly feel that the territoriality of knowledge (Huo et al., 2016) and malice of suspicion target and are more likely to hide knowledge. In contrast, employees with lower knowledge-based psychological ownership perceive less ownership and control of knowledge, and thus they less deliberately emphasize that knowledge is “mine” when facing the pressure of their own suspicion. Therefore, we presume the following hypothesis:

*Hypothesis 3*: Knowledge-based psychological ownership moderates the relationship between workplace suspicion and knowledge hiding, such that the relationship is stronger for employees with higher knowledge-based psychological ownership.

Thus far, we have explained how workplace suspicion leads to silence behavior *via* knowledge hiding and proposed that knowledge-based psychological ownership plays a moderating role in the relationship between workplace suspicion and knowledge hiding. According to the suggestion of Preacher et al. (2007), we further proposed a moderated mediation hypothesis that knowledge-based psychological ownership moderates the indirect effect of workplace suspicion on colleagues’ silence behavior *via* knowledge hiding. Since knowledge-based psychology intensifies the possibility of knowledge-hiding behavior caused by workplace suspicion, in the long run, the accumulation of knowledge-hiding behavior leads to an increase in the focus employee’s (i.e., the suspicion and hidden target) silence behavior. Therefore, we presume the following hypothesis:

*Hypothesis 4*: Knowledge-based psychological ownership moderates the indirect effect between workplace suspicion and silence behavior through knowledge hiding. Such an effect is more pronounced when knowledge-based psychological ownership is high rather than low.

### 2.4. The moderating role of face consciousness

Face consciousness refers to individuals’ desire to maintain, enhance, or avoid losing face with significant others in social interactions (Bao et al., 2003). As Zhang et al. (2011) suggested, face consciousness includes two correlated dimensions, namely, the desire to gain face and the fear of losing face. Specifically, “desire to gain face” reflects the individual’s desire to gain more social face, and “fear of losing face” reflects the individual’s fear of losing his or her existing social face (Zhang et al., 2011). The self-regulation theory holds that individual behavior is not only governed by his/her own subjective will but also affected by behavioral expectations and social expectations (Baumeister et al., 2005), so individuals are likely to adjust their behavior in response to social expectations. Face consciousness has motivational characteristics, and different levels of face consciousness could have important impacts on the subsequent cognition and action of individuals (Wang et al., 2019; Xu et al., 2022). Zheng et al. (2017) suggested that face consciousness could lead to employees’ desires for respect or recognition from their managers and colleagues, as well as concern about their own status and how others perceive them. Based on this, we infer that the influence of workplace suspicion on knowledge hiding would also be affected by face awareness.

From the perspective of “the desire for face,” individuals with a desire to gain face often yearn for social attention and recognition and tend to improve their fame through self-marketing and other means (Zhang et al., 2011; Jin et al., 2022). Knowledge, as a special personal possession, often provides a vehicle for the display and even enhancement of the face. In other words, it is an important way for employees to gain face by showing their ability or erudition and fully displaying their strengths and advantages through active knowledge sharing rather than knowledge hiding. Accordingly, compared with employees who have a low level of face consciousness, employees with a high level of face consciousness are more likely to adjust their expressive self-presentation (i.e., reduce rather than increase knowledge hiding) to maintain desired public appearances (Ho, 1976; Zhang et al., 2011; Xu et al., 2022), although workplace suspicion may make them worry about others’ requests for knowledge.

Additionally, from the “fear of losing face” perspective, employees with strong face consciousness are under pressure to live up to others’ expectations in order to maintain face (Gong et al., 2015; Zhao et al., 2019), and they worry naturally more about the loss of face (Chow et al., 1999). If an employee intentionally exposes their ignorance of knowledge when facing a knowledge request, they are easily afraid that they could be considered lacking knowledge and thus engage less in knowledge hiding (Zhao et al., 2019). Therefore, in the face of workplace suspicion, although employees with a high face consciousness may still hide knowledge, they have a stronger motivation to choose to reduce knowledge hiding as much as possible to avoid losing face. Conversely, the pain of “losing face” of employees with low face consciousness is lower than that of employees with high face consciousness (Xu et al., 2022), so they have a relatively weak tendency to deliberately suppress knowledge hiding when they suspect their colleagues.

In sum, compared with employees with low face consciousness, employees with high face consciousness will give priority to the gain and loss of face and adjust their behavior out of face consideration, no matter how high or low the level of suspicion in the workplace. Therefore, we presume the following hypothesis:

*Hypothesis 5*: Face consciousness moderates the relationship between workplace suspicion and knowledge hiding, such that the relationship is weaker for employees with higher face consciousness.

Combined with hypotheses 2 and 5, it can be further speculated that workplace suspicion indirectly promotes the occurrence of colleagues’ silence behavior through knowledge hiding, and the indirect effect depends on the level of employees’ facial awareness. Therefore, we presume the following hypothesis:

*Hypothesis 6*: Face consciousness moderates the indirect effect between workplace suspicion and silence behavior through knowledge hiding. Such an effect is more pronounced when face consciousness is low rather than high.

## 3. Materials and methods

### 3.1. Participants and procedures

The data were collected from 23 companies in eastern and southern China, including Shanghai, Nanchang, Guangzhou, Jinan, and other cities. These companies are mainly engaged in education, training, business consulting, machinery manufacturing, and other industries. The research takes the form of offline research, and the specific sampling process is as follows: first, the research team contacts the subjects who may participate in the research with the help of relationships, informs them of the form and purpose of the research, and makes a commitment to the subjects that the data is only used for academic research. Second, after obtaining the permission of the subjects, each subject is asked to determine 2–4 colleagues who have more contact with them in the same work team, and the investigator randomly invites one of those colleagues (i.e., focus employees) to conduct research, so as to finally form the data of the “employee-colleague” pairing. Furthermore, we committed all respondents regarding the confidentiality of the data, asked them to remain relaxed while filling out the questionnaire, and assured them that there is no correct or incorrect answer. As a result, as many natural answers as possible were obtained. To reduce the potential biases of the common method, the data collection procedure was completed in three phases, each separated by 40 days. In phase 1, initial subjects were required to assess their suspicion of peers, their level of knowledge-based psychological ownership, and face consciousness. In phase 2, initial subjects were asked to answer questions about knowledge hiding. In phase 3, the selected colleagues reported silence behavior and demographic characteristics.

In phase 1 of the survey, a total of 368 questionnaires were distributed to the employees, and 341 (92.66%) questionnaires were completed and returned. In phase 2, we requested these 341 respondents to answer the questionnaires and acquired 324 (88.04%) valid employee questionnaires. In phase 3, colleagues corresponding to employees in phase 2 were required to assess related questionnaires, and 303 colleagues returned their completed surveys. Finally, 303 “employee-colleague” matching questionnaires were formed, with a valid recovery rate of 82.33% (after excluding invalid questionnaires such as incomplete answers and irregular answers). Of the 303 corresponding targets, 178 (58.75%) are males and 125 (41.25%) are females, and the average age was 30.66 years. Moreover, regarding educational background, most of them have a bachelor’s degree (66.01%) or a master’s degree or above (20.13%), and the remaining 13.86% have a junior college education or below. Among them, most are general employees (73.9%), and managers are about 26.1%.

### 3.2. Measures

The measures were translated into Chinese and went through translation-back-translation procedures (Brislin, 1970) to verify the questionnaire in Chinese. The research used measuring instruments from prior studies, and the responses for all items ranged from 1 (“strongly disagree”) to 5 (“strongly agree”) on a Likert scale, except for the control variables such as gender, age, education, and position.

#### 3.2.1. Workplace suspicion

Using the measurement method of Bellou and Gkorezis (2016) for reference, we adapted the five-item scale to assess workplace suspicion developed by Bobko et al. (2014). The sample items included “In the process of interacting with a colleague, I become more and more suspicious.” Cronbach’s alpha for this scale was 0.83. (i.e., answered by suspicion perceivers).

#### 3.2.2. Knowledge hiding

The knowledge-hiding scale, including three items, was adapted by Peng (2013) for the Chinese context. Sample items included “I always withhold helpful information or knowledge from others.” Cronbach’s alpha calculated for this scale was 0.85. (i.e., answered by suspicion perceivers).

#### 3.2.3. Silence behavior

We used a five-item scale designed by Tangirala and Ramanujam (2008) to assess silence behavior. Sample items included “Although I have ideas or suggestions to improve my work, I do not say them.” Cronbach’s alpha calculated for this scale was 0.90. (i.e., answered by suspicion targets).

#### 3.2.4. Knowledge-based psychological ownership

Following the previous research (Peng, 2013), we asked employees to evaluate knowledge-based psychological ownership using a short, three-item version of the scale created by Dyne and Pierce (2004). The sample items included “I feel a very high degree of personal ownership of the knowledge.” The scale’s reliability was 0.88. (i.e., answered by suspicion perceivers).

#### 3.2.5. Face consciousness

Face consciousness was measured with 11 items developed by Zhang et al. (2011). The sample items included “I hope people think that I can do better than most others.” Cronbach’s alpha was 0.90. (i.e., answered by suspicion perceivers).

#### 3.2.6. Control variables

We controlled suspicion targets’ gender, age, education, and level of position as demographic variables, which have been shown to influence silence behavior (Tangirala and Ramanujam, 2008; Rai and Agarwal, 2018). Gender was coded as 0 = male and 1 = female. Age was coded 1 = 25 or below, 2 = 26–35, 3 = 36–45, and 4 = 46 or above. Education was coded 1 = vocational school or under, 2 = university, and 3 = graduate school. The level of position was coded 1 = general staff, 2 = low-level managers, 3 = middle managers, and 4 = senior managers.

### 3.3. Validity analyses

This study used several diagnostic analyses for addressing the common method bias. First, as previously mentioned, the data collection procedure was completed using a time lag approach. Second, the Harman monofactor analysis test was used to analyze the common method biases of the sample data, and the unrotated monofactor interpretation variable was 31.88%, not accounting for half of the total variance explained. Third, the one-factor model provided a poor fit [χ^2^(df) = 1568.39 (120), χ^2^/df = 13.07, *p* < 0.01; CFI = 0.55; TLI = 0.48; IFI = 0.55; RMSEA = 0.20], which indicated that the common method bias was not a serious threat in this study.

With regard to the rationality of the data structure, confirmatory factor analyses (CFA) using AMOS 24.0 were conducted to test the discriminant validity of the five constructs, namely, workplace suspicion, knowledge hiding, silence behavior, knowledge-based psychological ownership, and face consciousness. The discriminant validity of each scale was tested by comparing χ^2^(df), χ^2^/df, Δχ^2^(Δdf), CFI, TLI, IFI, and RMSEA (see Table 1). It is generally believed that an ideal model is proved if 1 < χ^2^/df < 3, CFI > 0.90, TLI > 0.90, IFI > 0.90, and RMSEA < 0.08 (Bentler and Bonett, 1980).

**Table 1:** The results of confirmatory factor analysis.

Measurement models		*χ^2^(df)*	*Δχ^2^(Δdf)*	χ^2^/df	*CFI*	*TLI*	*IFI*	*RMSEA*
Five-factor	WS, KH, SB, KPO, FC	291.35(108)^**^	-	2.70	0.94	0.93	0.94	0.07
Four-factor	WS + FC, KH, SB, KPO	557.98(113)^**^	266.63(5)^**^	4.94	0.80	0.83	0.86	0.11
Four-factor	WS + KPO, KH, SB, FC	828.40(113)^**^	537.05(5)^**^	7.33	0.72	0.73	0.78	0.15
Three-factor	WS + FC, KH + SB, KPO	848.46(116)^**^	557.11(8)^**^	7.31	0.72	0.73	0.77	0.15
Three-factor	WS + KPO, KH + SB, FC	1119.84(116)^**^	828.49(8)^**^	9.65	0.69	0.63	0.69	0.17
Two-factor	WS + KPO + KH, SB + FC	1170.21(118)^**^	878.86(10)^**^	9.92	0.67	0.62	0.67	0.17
Two-factor	WS + FC + KH, SB + KPO	1205.19(118)^**^	913.84(10)^**^	10.21	0.64	0.61	0.66	0.18
One-factor	WS + KH + SB + KPO + FC	1568.39(120)^**^	1277.04(12)^**^	13.07	0.55	0.48	0.55	0.20

WS, workplace suspicion; KH, knowledge hiding; SB, silence behavior; KPO, knowledge-based psychological ownership; FC, face consciousness. “+” means that two factors are combined into one factor. ^**^p < 0.01.

As shown in Table 1, the expected five-factor model (workplace suspicion, knowledge hiding, silence behavior, knowledge-based psychological ownership, and face consciousness) provided a reasonable fit to the data [χ^2^(df) = 291.35 (108), χ^2^/df = 2.70, *p* < 0.01; CFI = 0.94; TLI = 0.93; IFI = 0.94; RMSEA = 0.07]. In addition, we measured seven alternative models with different combinations of focal variables. The results reported that our expected model had significantly better fitted the data than the alternative models. Moreover, according to Bentler and Bonett (1980), we also used χ^2^ difference (Δχ^2^) to determine the best-fitting model. The results indicated that the baseline five-factor model was significantly improved compared with the four-factor model [Δχ^2^(5) = 266.63 or 537.05, *p* < 0.01]; the three-factor model [Δχ^2^(8) = 557.11 or 828.49, *p* < 0.01]; the two-factor model [Δχ^2^(10) = 878.86 or 913.84, *p* < 0.01]; and the single-factor model [Δχ^2^(12) = 1277.04, *p* < 0.01], suggesting that the five focal variables could be clearly distinguished.

Besides, we used CR and AVE to evaluate the convergent validity of all variables. The composite reliability of all five constructs exceeds 0.70, and the AVE values of all constructs exceed 0.50. These reveal that convergent validity is good. In addition, the discriminative validity was further verified. The square roots of all variables’ AVE values are larger than 0.70, exceeding the correlations of all variables in Table 2. Therefore, all constructs have adequate consistent and discriminant validity. In conclusion, the results of the validity analysis show that the data construct is clear and suitable for correlation analysis.

**Table 2:** Descriptive statistical results and correlation coefficients matrix.

	1	2	3	4	5	6	7	8	9
Gender	−								
Age	0.08	−							
Education	0.04	0.05	−						
Level of position	−0.01	0.57^**^	−0.04	−					
WS	−0.01	0.07	0.03	−0.06	**0.83**				
KH	−0.09	0.03	−0.06	−0.04	0.46^**^	**0.85**			
SB	−0.05	−0.08	−0.06	−0.14^*^	0.34^**^	0.55^**^	**0.90**		
KPO	−0.02	0.07	0.01	0.02	0.28^**^	0.47^**^	0.33^**^	**0.88**	
FC	0.04	0.05	−0.02	0.10	−0.40^**^	−0.59^**^	−0.53^**^	−0.55^**^	**0.90**
M	1.59	30.66	2.06	1.34	3.07	2.67	2.89	3.35	2.88
SD	0.49	5.59	0.58	0.64	0.47	0.92	0.86	0.82	0.71

*N* = 303. WS, workplace suspicion; KH, knowledge hiding; SB, silence behavior; KPO, knowledge-based psychological ownership, FC, face consciousness; M, mean; SD, standard deviation. ^**^p < 0.01, ^*^p < 0.05. Cronbach’s α reliability coefficients appear on the diagonal.

## 4. Results

### 4.1. Descriptive statistics and correlation analysis

Table 2 shows the means, standard deviations, and correlations of the variables. In line with our expectation, workplace suspicion was positively related to silence behavior (*r* = 0.34, *p* < 0.01) and significantly affected knowledge hiding (*r* = 0.46, *p* < 0.01); knowledge hiding had a positive relationship with silence behavior (*r* = 0.55, *p* < 0.01). Hypothesis 1 was initially supported and provides a basis for further analysis.

### 4.2. Mediation results

Table 3 presents the results, analyzed by SPSS 26.0 and the PROCESS program developed by Hayes and Preacher (2014), for the mediated and moderated effects of the model. We used model 4 of the PROCESS program to test the mediation effect. The result revealed that workplace suspicion is positively associated with silence behavior (β = 0.60, *p* < 0.01) supports hypothesis 1. When we add knowledge hiding into the model as a mediator, we found that the direct effect of workplace suspicion on silence behavior was not significant (β = 0.19, n.s.) but the indirect effect was (β = 0.60, *p* < 0.01). Moreover, the indirect effect accounted for 68.33% of the total effect, which indicates that knowledge hiding mediated the influence of workplace suspicion on silence behavior. In addition, bootstrapping procedures were used to construct the confidence interval (CI) in estimating the mediating effect. As can be seen from the result, the indirect effect of workplace suspicion on silence behavior *via* knowledge hiding was also significant (95% CI [0.26, 0.58], excluding 0). Consequently, hypothesis 2 was supported.

**Table 3:** Conditional process analysis: mediation and moderation results.

	Effect	SE	Boot 95% CI		Proportion of effect
			LL	UL	
Total effect: WS → SB	0.60^**^	0.10	0.41	0.80	
**The mediating effect test**					
Direct effect: WS → SB	0.19	0.12	−0.07	0.41	31.67%
Indirect effect: WS → KH → SB	0.41^**^	0.08	0.26	0.58	68.33%

**The moderating effect test**					
The moderating results		Effect	SE	Boot 95%CI	
				LL	UL
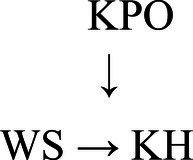	Low (M − 1 SD)	0.51^**^	0.12	0.27	0.74
	Mean(M)	0.70^**^	0.09	0.52	0.88
	High (M + 1 SD)	0.89^**^	0.11	0.67	1.11
	Interaction	0.23^*^	0.10	0.04	0.43
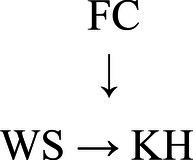	Low (M − 1 SD)	0.78^**^	0.12	0.54	1.03
	Mean(M)	0.55^**^	0.09	0.36	0.73
	High (M + 1 SD)	0.31^**^	0.11	0.09	0.53
	Interaction	−0.34^**^	0.10	−0.53	−0.14

**The moderated mediation effect test**					
The bootstrapping results of conditional indirect effect		Effect	SE	Boot 95%CI	
				LL	UL
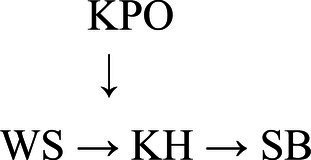	Low (M − 1 SD)	0.25^**^	0.08	0.10	0.40
	Mean(M)	0.33^**^	0.07	0.19	0.47
	High (M + 1 SD)	0.41^**^	0.08	0.25	0.58
	The results of moderated mediation	0.10^*^	0.05	0.01	0.19
	Low (M − 1 SD)	0.35^**^	0.08	0.20	0.51
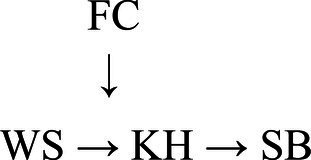	Mean(M)	0.25^**^	0.06	0.13	0.38
	High (M + 1 SD)	0.15^*^	0.07	0.03	0.29
	The results of moderated mediation	−0.14^**^	0.05	−0.24	−0.05

WS, workplace suspicion; KH, knowledge hiding; SB, silence behavior; KPO, knowledge-based psychological ownership; FC, face consciousness. Bootstrapping sample size = 5,000, *N* = 303, ^**^p < 0.01, ^*^p < 0.05.

### 4.3. Moderation results

**Figure 2:**
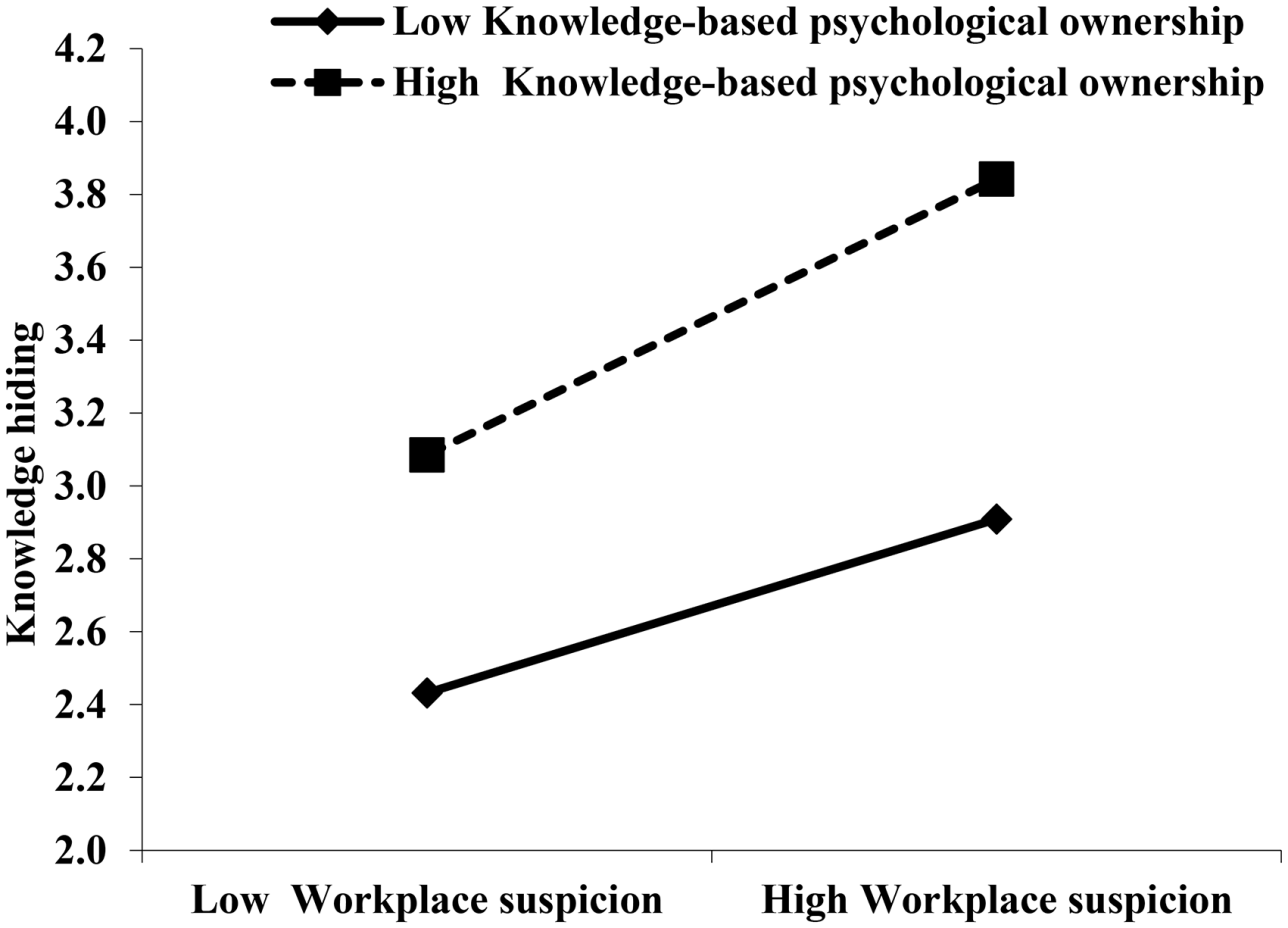
The moderating effect of knowledge-based psychological ownership on the relationship between workplace suspicion and knowledge hiding.

We used model 1 of the PROCESS program to test the moderation effect (hypotheses 3 and 5). Hypothesis 3 predicts that knowledge-based psychological ownership moderates the relationship between workplace suspicion and knowledge hiding, such that the relationship is stronger for employees with higher knowledge-based psychological ownership. Consistent with this hypothesis, the results in the middle of Table 3 reveal that the interaction of workplace suspicion with knowledge-based psychological ownership had a significant impact on knowledge hiding (β = 0.21, *p* < 0.05). Furthermore, to obtain a more intuitive response, the interaction effects of knowledge-based psychological ownership or face consciousness at different levels (i.e., −1 SD and +1 SD), we plotted the moderating effect figures separately according to the suggestions of Cohen et al. (1985). Figure 2 reveals that the impact of workplace suspicion on knowledge hiding was significant when knowledge-based psychological ownership was high (effect size = 0.87, *p* < 0.01) rather than low (effect size = 0.53, *p* < 0.01). Thus, hypothesis 3 was supported.

**Figure 3:**
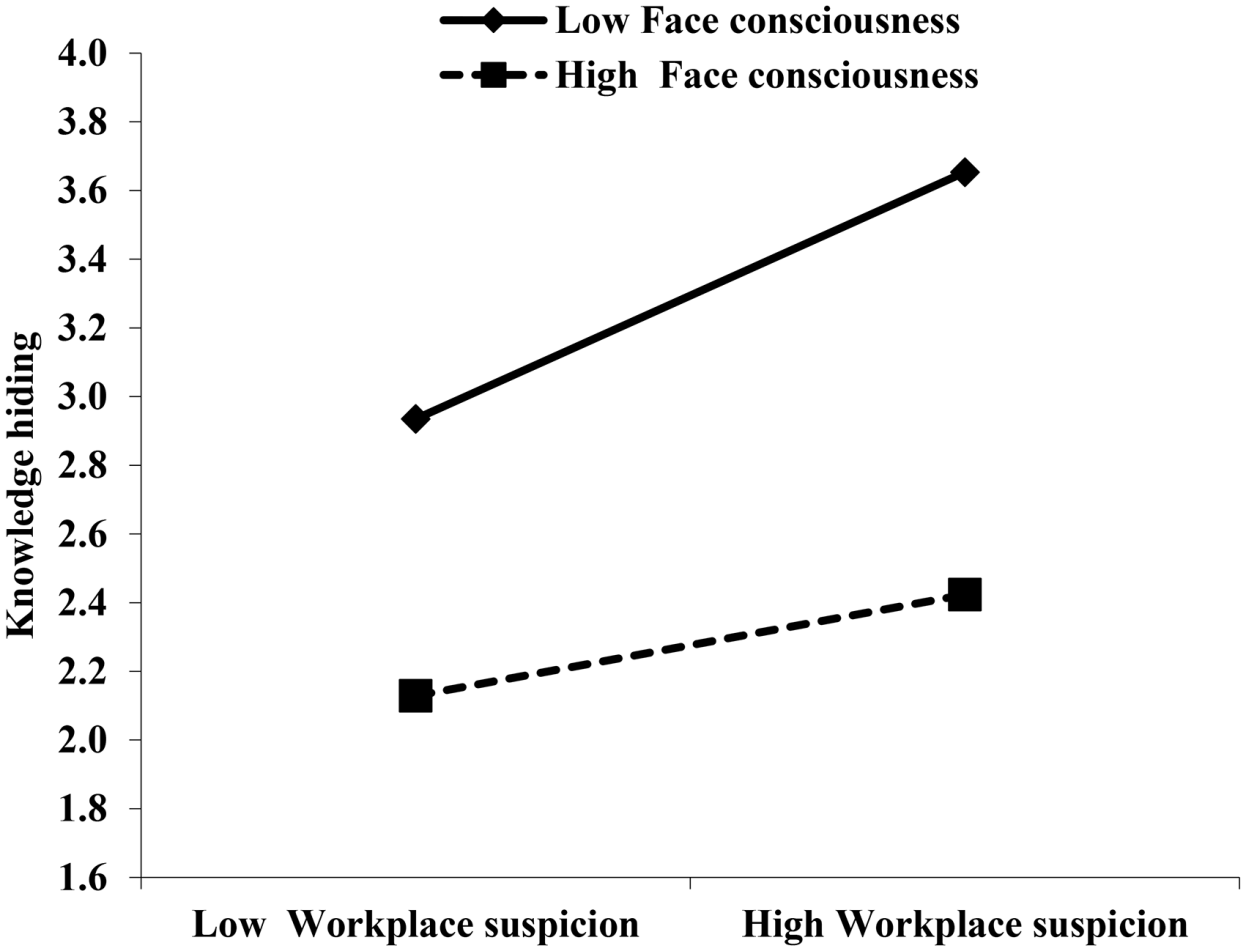
The moderating effect of face consciousness on the relationship between workplace suspicion and knowledge hiding.

Hypothesis 5 predicts that face consciousness moderates the relationship between workplace suspicion and knowledge hiding, such that the relationship is weaker for employees with higher face consciousness. The results showed that the interaction of workplace suspicion with face consciousness was significantly related to knowledge hiding (β = −0.30, *p* < 0.01). As shown in Figure 3, the impact of workplace suspicion on knowledge hiding was more significant when face consciousness was low (effect size = 0.76, *p* < 0.01) rather than high (effect size = 0.33, *p* < 0.01). These findings supported hypothesis 5.

Furthermore, we applied model 7 of the SPSS PROCESS macro to test the moderated mediation effects (hypotheses 4 and 6). Hypothesis 4 assumes that knowledge-based psychological ownership moderates the indirect effect between workplace suspicion and silence behavior through knowledge hiding and that such an effect is more pronounced when knowledge-based psychological ownership is higher. Following Hayes (2015), we first analyzed the index of moderated mediation, which provides a statistically more formal test than testing the indirect relationships at high and low values of moderator. The index of moderated mediation was significant (effect size = 0.10, *p* < 0.05), which indicated that hypothesis 3 was supported. Moreover, as shown in Table 3, the indirect effect of workplace suspicion on silence behavior through knowledge hiding was more positive (effect size = 0.41, *p* < 0.01) at a high level (i.e., +1 SD) of knowledge-based psychological ownership than at a low level (i.e., −1 SD) of knowledge-based psychological ownership (effect size = 0.25, *p* < 0.01). Therefore, these findings provided support for hypothesis 4.

We then examined face consciousness as a moderator. In hypothesis 6, the indirect effect between workplace suspicion and silence behavior through knowledge hiding obscures the focus on the moderating role. Consistent with hypothesis 6, the index of moderated mediation was negative and significant (effect size = −0.14, *p* < 0.01). The indirect effect of workplace suspicion on silence behavior through knowledge hiding was more positive (effect size = 0.35, *p* < 0.01) at a low level (i.e., −1 SD) of knowledge-based psychological ownership than at a high level (i.e., +1 SD) of knowledge-based psychological ownership (effect size = 0.15, *p* < 0.05). In other words, high face consciousness has a stronger inhibition of the indirect effect. These findings lend support to hypothesis 6.

## 5. Discussion

Based on the conservation of resources theory and self-regulation theory, this study explained how and when workplace suspicion may lead to colleagues’ silence behavior. We developed and studied a double-moderated mediation model in which the relationship between workplace suspicion, knowledge hiding, and silence behavior is moderated by knowledge-based psychological ownership and face consciousness. This study found that workplace suspicion is a negative phenomenon in the workplace that can deplete both suspicion perceivers’ and suspicion targets’ resources. Specifically, workplace suspicion is positively correlated with silence behavior; knowledge hiding mediates the relationship between workplace suspicion and silence behavior; knowledge-based psychological ownership moderates this mediating effect by strengthening the negative impact of workplace suspicion on knowledge hiding; and face consciousness moderates the mediating effect by weakening the positive impact of workplace suspicion on knowledge hiding. This study provided a new idea for the organization on how to prevent and control knowledge-hiding and silence behaviors from the perspective of colleagues. Managers and practitioners are suggested to consider the phenomenon of workplace suspicion and the focus on employees’ knowledge-based psychological ownership and face consciousness when working to weaken knowledge-hiding and silence behavior.

### 5.1. Theoretical implications

This study provides several theoretical implications. First, the study contributes to silence behavior literature by examining workplace suspicion as an antecedent variable of a colleague’s perspective. Some scholars have explored the antecedents of employees’ silence behavior from the perspective of individuals, leaders, and organizations (e.g., Wynen et al., 2020; Wan et al., 2021; Wei et al., 2022). However, as an important source of information in the organization (Chen et al., 2021; Jin et al., 2022), the influence of colleagues’ behavior (especially suspicious behavior) on employees’ silence behavior has been ignored by previous studies. Based on this, we explore how workplace suspicions affect colleagues’ silence behavior from a binary perspective.

Second, the research advances the current understanding of workplace suspicion by theoretically proposing and empirically testing its negative consequences. Suspicion is a widespread and influential phenomenon in organizations, but relevant empirical research is very limited (Bobko et al., 2014; Zhou et al., 2017). The behavioral responses of suspicion perceivers and suspicion targets were investigated, respectively. We established a correlation between workplace suspicion and silence behavior as well as analyzed the underlying mechanisms between the two through the behavior of suspicion perceivers. This research responds to the call of scholars (e.g., Bobko et al., 2014) for more research on suspicion in the field of organizational behavior.

Third, from a COR theory perspective, this article reveals that knowledge hiding provides a unique and novel theoretical account for the effects of workplace suspicion combined with individual factors on colleagues’ silence behavior. He et al. (2021) pointed out that it is essential to increase research on the consequences of knowledge hiding to enrich the antecedents’ knowledge-hiding consequences research path, and our study responds to this call. The research on the intermediary mechanism of knowledge hiding extends Bari et al.’s (2020) study that found a positive relationship between knowledge hiding and employee silence and provides a new theoretical basis for the related research on knowledge hiding and silence behavior.

Finally, drawing on the conservation of resources theory and self-regulation theory, the double boundary conditions at the individual level are verified in the process of workplace suspicion of knowledge hiding. In the previous studies on knowledge hiding, numerous researchers have paid attention to the role of knowledge-based psychological ownership, and our findings are basically consistent with them. In addition, the study responds to the call of some scholars that consciousness has a universal nature that ought to be extended to a myriad of further research areas (Kim and Nam, 1998; Zhang et al., 2011). In short, this study clarifies the moderating conditions for the negative effects of workplace suspicion from the perspective of individual differences, which provides further evidence for the situational behavior of colleagues’ silence and also enriches the literature on knowledge-based psychological ownership and face consciousness.

### 5.2. Practical implications

Our findings offer several managerial implications. First of all, workplace suspicion will positively exacerbate knowledge hiding and colleagues’ silence behavior. Therefore, active measures should be taken by organizations to restrain workplace suspicion. For instance, when the teams recruit employees, it is necessary to properly test the suspicion tendency of candidates and to reduce the appointment of individuals with excessive suspicion. Besides, managers need to care about the real thoughts of individuals and give employees some opportunities (e.g., a team-building activity) to allow them to know each other. The sense of mutual trust among employees, especially marginalized workers (e.g., new employees), should be cultivated and enhanced.

Second, our results support that knowledge hiding plays a dominant role in mediating the relationship between workplace suspicion and silence behavior. Therefore, organizations should build a working environment that is filled with knowledge sharing rather than knowledge hiding and reduce risks from colleagues through employee voice. Furthermore, combined with the scholars’ (e.g., He et al., 2021) theoretical viewpoint that organizational atmosphere can alleviate the negative effect of knowledge hiding, this article suggests that a knowledge-sharing atmosphere and a learning atmosphere should be created within the organization to encourage staff knowledge exchange and suppress the negative effect of staff knowledge hiding.

Finally, our results support the idea that knowledge-based psychological ownership and face consciousness are important in moderating the relationship between workplace suspicion and knowledge hiding. Accordingly, we put forward some suggestions for organizations and practitioners. On the one hand, managers must attach importance to knowledge-based psychological ownership. In practice, it is feasible for organizations to boost employees’ team awareness and cooperation, which helps them claim their knowledge as “ours.” Organizations should guide employees to reduce their sense of territorial protection in knowledge sharing (Huo et al., 2016) and strive to make employees realize that sharing knowledge with others will not make them lose their advantages but can increase each other’s knowledge stocks through “reciprocity” to achieve win-win results. On the other hand, practitioners need to pay attention to employees’ facial consciousness. For example, we recommend that organizations give full play to the role of spiritual motivation when designing the incentive system. More specifically, organizations can adopt the methods of honor motivation and responsibility motivation to give more recognition and respect to employees who have made certain knowledge-sharing contributions, give them greater rights and responsibilities, and make face consciousness the eliminator for knowledge hiding.

### 5.3. Limitations and future research directions

This research certainly has some limitations. First, considering that the focus of this study is on the behaviors themselves, we did not separate their dimensions to examine whether workplace suspicion has differential effects on them. However, it can be considered to explore the specific relationships and mechanisms between different dimensions of these variables in more detail. A future study could develop a comprehensive model by considering multiple dimensions of these variables. Second, although we designed a three-stage time-lag study to attempt to establish the causality of variables, the proposed causality may not be fully determined since the data were essentially relevant. Besides, although we controlled the suspicion targets’ gender, age, education, and level of position factors that may affect silence behavior, there are also other important factors. For these reasons, a longitudinal, experimental design or the addition of important control variables would be ideal for future research to improve the robustness of research conclusions. Third, the study merely examined the moderating roles at individual levels (i.e., knowledge-based psychological ownership and face consciousness). In the future, other personality traits (e.g., neuroticism) and situational moderators (e.g., organizational ethical climate) may be considered alternative boundary conditions.

## Data availability statement

The raw data supporting the conclusions of this article will be made available by the authors, without undue reservation.

## Author contributions

MW and WL: conceptualization and validation. MW: methodology and software. WL and LZ: formal analysis. LZ: investigation. CZ: resources. MW: data curation, supervision, and funding acquisition. WL: writing—original draft preparation. WL and HZ: writing—review and editing. MW and CZ: project administration. All authors contributed to the article and approved the submitted version.

## Funding

This research was funded by Philosophy and Social Science Foundation of Shanghai (CN) under grant number (2019EGL011).

## Conflict of interest

The authors declare that the research was conducted in the absence of any commercial or financial relationships that could be construed as a potential conflict of interest.

## Publisher’s note

All claims expressed in this article are solely those of the authors and do not necessarily represent those of their affiliated organizations, or those of the publisher, the editors and the reviewers. Any product that may be evaluated in this article, or claim that may be made by its manufacturer, is not guaranteed or endorsed by the publisher.

## Figures and Tables

**Table:** 

Construct	Measuring items
Workplace suspicion (WS) (Bobko et al., 2014)	WS1 In the process of interacting with a colleague, I become more and more suspicious.
	WS2 I was on my guard when interacting with my colleagues.
	WS3 I was not suspicious about what was being presented to me. (S, reverse scored)
	WS4 I am not suspicious of anything that happens. (S, reverse scored)
	WS5 I am suspicious of my colleagues.
Knowledge hiding (KH) (Peng, 2013)	KH1 I always withhold helpful information or knowledge from others.
	KH2 I always try to hide innovative achievements.
	KH3 I do not transform personal knowledge and experience into organizational knowledge.
Silence behavior (SB) (Tangirala and Ramanujam, 2008)	SB1 I choose to remain silent when I have concerns about the work.
	SB2 Although I have ideas for improving the work, I do not speak up.
	SB3 I say nothing to others about the problems I notice in my workgroup.
	SB4 I remain silent when I have information that might help prevent an incident in my workgroup.
	SB5 I keep quiet instead of asking questions when I want to get more information about the work.
Knowledge-based psychological ownership (KPO) (Peng, 2013)	KPO1 This is my knowledge. KPO2 I feel a very high degree of personal ownership of the knowledge. KPO3 I sense that this is my knowledge.
Face consciousness (FC) (Zhang et al., 2011)	FC1 I hope people think that I can do better than most others.
	FC2 I hope that I can talk about things that most others do not know.
	FC3 I hope that I can possess things that most others thirst for.
	FC4 It is important for me to get praise and admiration.
	FC5 I hope to let people know that I have association with some big names.
	FC6 I hope that I have a better life than most others in others’ view.
	FC7 I always avoid talking about my weakness.
	FC8 I try to avoid letting others think that I am ignorant, even if I really am.
	FC9 I do my best to hide my weakness before others.
	FC10 If I work in an organization of bad reputation, I will try not to tell others about that.
	FC11 It is hard for me to acknowledge a mistake, even if I am really wrong.

## Appendix

Appendix 1. Questionnaire items.
